# Connections between job satisfaction and depression, anxiety, and stress among nurses

**DOI:** 10.3389/fpsyg.2025.1548993

**Published:** 2025-02-05

**Authors:** Agnė Buivydienė, Lolita Rapolienė, Marija Truš, Agnė Jakavonytė-Akstinienė

**Affiliations:** Department of Nursing, Faculty of Health Sciences, Klaipėda University, Klaipėda, Lithuania

**Keywords:** anxiety, depression, job satisfaction, nurse, stress

## Abstract

**Introduction:**

As a cornerstone of overall wellbeing, mental health significantly influences job satisfaction, vital for employee retention and organizational success. Recognizing this, managers prioritize initiatives to enhance job satisfaction and promote a healthy, productive work environment.

**Aims:**

The study aims to explore the connections between depression, anxiety, stress, and job satisfaction among nurses, providing insights to improve their wellbeing and retention in the profession.

**Methods:**

This quantitative survey study was conducted from June to September 2024, involving 643 nurses from healthcare facilities in Klaipėda County. Data were collected using two validated instruments: the Depression, Anxiety Stress Scales (DASS-42) and the Paul Spector Job Satisfaction Survey (JSS). Statistical analysis was performed using IBM SPSS Statistics Version 29.0.1.0.

**Results:**

The study found that while the average levels of depression, anxiety, and stress among nurses were within normal limits, a significant portion of participants exhibited compromised mental health. Nearly one-third of the nurses experienced stress and/or depression, and almost half reported anxiety. Over one-sixth of the participants experienced high or very high levels of anxiety, while severe depression or stress was less common. Factors such as years of experience, workload, and work schedule were identified as significant influences on nurses’ psychoemotional state. Moderate to strong positive correlations were observed between depression, anxiety, and stress levels. Additionally, the nurses’ mental health was statistically significantly and negatively associated with most components of job satisfaction.

**Conclusion:**

Despite average levels of depression, anxiety, and stress being within normal limits, many nurses face significant mental health challenges, particularly anxiety. Factors like workload and work schedules strongly influence their wellbeing. The negative link between mental health and job satisfaction highlights the need for better support to improve nurses’ wellbeing and job satisfaction.

## 1 Introduction

Job satisfaction reflects an employee’s attitudes and feelings toward their work, encompassing various aspects such as working conditions, compensation, job content, and relationships with colleagues and supervisors. This subjective evaluation significantly influences motivation, productivity, and wellbeing. Job satisfaction can alleviate stress, enhance emotional health, and improve life quality for employees. It translates into lower turnover rates, higher productivity, improved work quality, and a positive organizational climate for organizations. Furthermore, satisfied employees are likelier to demonstrate loyalty and commitment, driving long-term organizational performance and competitive advantage (Lu et al., 2019). Promoting job satisfaction through mental health requires a holistic approach. Internally, building psychological capital can strengthen employees’ resilience and self-efficacy, while externally, fostering social capital through supportive relationships and networks enhances their sense of belonging and security (Cao et al., 2022). In the workplace, mental health is pivotal in shaping job satisfaction, a key factor in employee retention and organizational success (Warszewska-Makuch, 2021). Managers increasingly recognize that fostering job satisfaction is essential for maintaining a healthy and productive workforce (Tang et al., 2019). Poor mental health disrupts one’s ability to lead an active and fulfilling life and is often accompanied by conditions such as depression, anxiety, and fear (Palinkas and Wong, 2020). This issue is particularly critical in healthcare professions, where workers face intense mental and physical demands. Factors such as long working hours, rotating shifts, exposure to human suffering, and workplace violence contribute to heightened psychological stress (Starc, 2018; Bautista et al., 2020). As frontline healthcare providers, nurses experience additional challenges due to their close contact with patients and the high workload associated with constantly changing, stressful situations (Butler et al., 2018; Nigam et al., 2023). Psychologically safe work environments have been associated with better worker wellbeing (Lu et al., 2019). Job dissatisfaction among nurses, fueled by workplace stress and anxiety, not only affects their performance but also drives some to leave their positions or even the profession altogether (Geese et al., 2022). Globally, the healthcare sector faces a severe human resource crisis, with a shortage of over 5.7 million nurses projected to persist for at least the next decade (World Health Organization, 2024). Addressing this challenge requires identifying the elements of mental health that influence various aspects of job satisfaction. This study aims to explore the connections between depression, anxiety, stress, and job satisfaction among nurses, providing insights to improve their wellbeing and retention in the profession.

## 2 Materials and methods

### 2.1 Study design and setting

The study was designed as a cross-sectional survey with self-reported questionnaires. The study was conducted between June and September 2024. The study was conducted in primary healthcare institutions and facilities providing inpatient healthcare services in Klaipėda County.

The research adhered to the fundamental ethical principles in the Declaration of Helsinki. It was approved by the Ethics Committee of the Nursing Department, Faculty of Health Sciences, Klaipėda University, on March 14, 2024 (Approval No. 46Sv–S–14).

### 2.2 Participants

A non-probability purposive sampling method was applied to enhance sample representativeness. Using Paniotto’s formula, a sample size of 643 participants was determined to ensure a 5% margin of error and 95% confidence level. Participants included nurses working in Klaipėda County, employed in institutions offering outpatient and inpatient healthcare services, proficient in the Lithuanian language, and voluntarily agreeing to participate.

### 2.3 Data collection

Two validated questionnaires were used for data collection: the Depression Anxiety Stress Scales (DASS-42) and the Paul Spector Job Satisfaction Survey (JSS).

#### 2.3.1 DASS-42

The self-report scale designed to measure the negative emotional states of depression, anxiety, and stress in adults and older adolescents comprises 42 items designed to measure individual levels of depression, anxiety, and stress (Lovibond and Lovibond, 1995). Each subscale contains 14 items: stress (items 1–14), anxiety (items 15–28), and depression (items 29–42). Scores were categorized as follows: total score: normal (0–32), mild (33–39), moderate (40–49), severe (50–57), and extremely severe (58+). Depression: normal (0–9), mild (10–13), moderate (14–20), severe (21–27), extremely severe (28+). Anxiety: normal (0–7), mild (8–9), moderate (10–14), severe (15–19), extremely severe (20+). Stress: normal (0–14), mild (15–18), moderate (19–25), severe (26–33), extremely severe (34+). The DASS-42 is widely used in clinical research, psychological evaluations, and healthcare practice to assess psychological wellbeing objectively. To assess the reliability of the DASS-42 scale in a study with nursing students, Cronbach's alpha coefficients were calculated separately for the whole scale and each subscale. The Cronbach's alpha coefficient for the full DASS-42 scale was 0.96. For each scale: depression 0.93, anxiety 0.89, and stress 0.92 (Makara-Studzińska et al., 2022). The Lithuanian version of the scale has sufficient reliability (Cronbach’s alpha = 0.75) and the overall internal reliability of the whole questionnaire (Cronbach’s alpha = 0.71) (Kuodytė-Kazielienė and Sondaitė, 2013).

#### 2.3.2 JSS

The Paul Spector Job Satisfaction Survey (JSS) questionnaire was used to evaluate job satisfaction. This tool consists of 36 items grouped into nine facets, designed to assess employees’ attitudes toward various aspects of their work, including pay, promotion, supervision, fringe benefits, contingent rewards, operating procedures, coworkers, nature of work, and communication. The total score ranges from 36 to 216, categorized as dissatisfaction (36–108), ambivalent (108–144), and satisfaction (144–216) (Spector, 1985). This questionnaire was translated and validated into Lithuanian by Gustainienė et al. (2009). The Paul Spector Job Satisfaction Scale had an overall coefficient alpha (based on a sample of 2,870) of 0.91 (Spector, 1985). In Lithuania, Leketas conducted a study in 2022 and found that the Cronbach’s alpha of The Paul Spector Job Satisfaction Scale was 0.862 (Leketas, 2022).

### 2.4 Data management and analysis

Statistical analysis was performed using MS Excel 2010 and IBM SPSS Statistics Version 29.0.1.0. Data were presented in tables and graphs, showing percentages, absolute distributions, means with standard deviations (M ± SD), medians, and minimum and maximum values. The Kolmogorov–Smirnov test assessed the normality of data distributions. Due to non-normal distributions, the Mann–Whitney U test was used for two-group mean comparisons, and the Kruskal–Wallis H test with pairwise comparisons was applied to identify significant differences among more than two groups. Statistical significance was set at *p* < 0.05.

## 3 Results

The study involved 643 respondents, the majority of whom were women (99.2%). The average age of the respondents was 42 years. More than half of the participants (60.2%) had obtained a higher non-university education. During the survey, most respondents had 1–5 years of work experience at their current workplace (25.5%). However, 133 respondents (20.7%) reported a total work experience of 31 years or more. Over half of the respondents worked part-time (0.6–1.0 full-time equivalent, 61.4%), in day shifts (52.3%), and for the majority (84.1%), this was their sole place of employment.

It was found that the total DASS-42 score was within normal limits (24.9), and none of the psychoemotional state scales reached the threshold for a disorder. The lowest average scores were observed on the depression subscale for nurses (6.4 ± 6.5), while the highest was on the stress subscale (10.6 ± 6.2), with an average anxiety score of 6.9 ± 6.

Based on scale values assessing the distribution of psychoemotional states among nurses, 73.1% had normal stress levels, 53.2% had normal anxiety levels, and 69.8% had normal depression levels (Table 1). The most significant disturbance was observed on the anxiety scale: 14.8% of respondents reported high or very high anxiety levels, whereas high levels of depression were observed in 2.4% of respondents and high-stress levels in 1.4%.

**Table 1:** Expression of nurses’ psychoemotional state.

Severity levels	Nurses (*n* = 643)		
	Depression	Anxiety	Stress
Normal	449 (69.8%)	342 (53.2%)	470 (73.1%)
Mild	58 (9.0%)	85 (13.2%)	95 (14.8%)
Moderate	121 (18.8%)	121 (18.8%)	69 (10.7%)
Severe	15 (2.4%)	55 (8.6%)	9 (1.4%)
Extremely severe	—	40 (6.2%)	—

**Figure 1:**
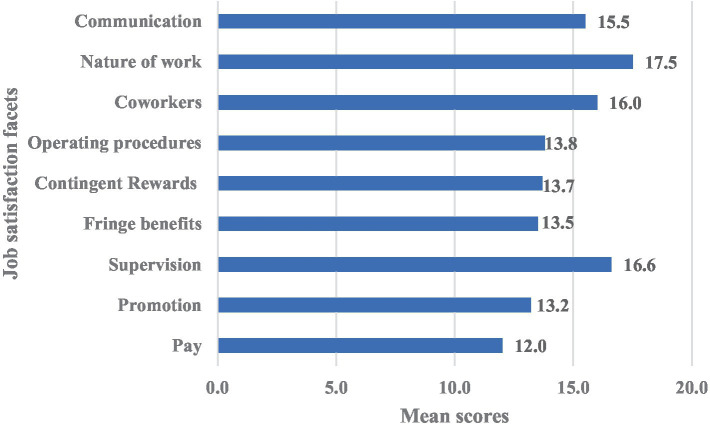
Results of nurses’ job satisfaction assessment, average scores.

On the overall satisfaction scale, nurses scored an average of 131.7 ± 20.0 points. It was found that the highest-rated aspects were the nature of the work (17.5 ± 4.0) and supervision, while the lowest-rated were salary (12.0 ± 3.6) and promotion (Figure 1).

Significant correlations were identified between psychoemotional state, job satisfaction, and sociodemographic factors. According to pairwise comparisons, higher levels of depression were observed among nurses working in outpatient settings with low workloads, multiple workplaces, and night shifts. More significant anxiety was reported by younger nurses (−0.119), males, those with less than 5 years of experience, low or high workloads, or those working night or 24-h shifts. Higher stress levels were found among nurses with 1–5 years of experience and those working low or high workloads on 24-h shifts (Table 2).

**Table 2:** Comparison of nurses’ psychoemotional state and job satisfaction based on their sociodemographic characteristics.

Parameters	Depression, mean (SD)	*p*	Anxiety, mean (SD)	*p*	Stress, mean (SD)	*p*	Job satisfaction, mean, SD	*p*
Age, year 42.0 (13.4)		*p* = 0.667*		*p* = 0.003*		*p* = 0.617*		*p* = 0.784*
Gender								
Female (*n* = 638)	4.0 (6.4)	*p* = 0.236**	7.0 (7.9)	*p* = 0.029**	10.0 (10.7)	*p* = 0.625**	128.0 (131.7)	*p* = 0.783**
Man (*n* = 5)	8.0 (7.2)		8.0 (10.0)		12.0 (9.7)		126.0 (128.9)	
Education								
University (*n* = 225)	4.0 (7.2)	*p* = 0.471***	8.0 (8.1)	*p* = 0.407***	11.0 (10.6)	*p* = 0.479***	129.0 (131.2)	*p* = 0.148***
High non-university (*n* = 387)	5.0 (6.0)		7.0 (7.7)		10.0 (10.6)		128.0 (132.0)	
Other (*n* = 5)	5.0 (5.9)		9.0 (8.6)		10.0 (11.4)		125.0 (131.2)	
Length of service in the last institution								
< 1 year (*n* = 76)	3.5 (5.6)	*p* = 0.208***	8.0 (7.4)	*p* = 0.062***	8.0 (8.7)	*p* = 0.031***	137.0 (139.6)	*p* < 0.001***
1–5 years (*n* = 162)	3.0 (5.8)		7.0 (8.9)		10.0 (11.6)		127.5 (128.1)	
6–10 years (*n* = 101)	6.0 (7.9)		5.0 (8.3)		10.0 (11.2)		125.0 (126.6)	
11–20 years (*n* = 131)	3.0 (6.3)		6.0 (6.6)		11.0 (9.8)		126.0 (132.4)	
21–30 years (*n* = 79)	2.0 (5.4)		7.0 (8.1)		11.0 (10.5)		128.0 (129.9)	
≥31 years (*n* = 94)	8.0 (7.7)		7.0 (7.7)		10.0 (11.3)		134.0 (137.4)	
Full length of service								
< 1 year (*n* = 53)	5.0 (7.5)	*p* = 0.107***	8.0 (9.2)	*p* < 0.001***	9.0 (10.0)	*p* = 0.040***	137.0 (135.7)	*p* = 0.013***
1–5 years (*n* = 130)	5.0 (6.4)		10.0 (10.4)		12.0 (12.6)		126.0 (125.6)	
6–10 years (*n* = 106)	2.0 (6.2)		5.0 (7.1)		8.5 (9.8)		127.0 (133.3)	
11–20 years (*n* = 122)	3.0 (6.4)		6.0 (6.8)		11.0 (9.9)		125.0 (133.3)	
21–30 years (*n* = 99)	2.0 (5.5)		6.0 (6.9)		10.0 (10.2)		130.0 (131.3)	
≥31 years (*n* = 133)	6.0 (6.9)		7.0 (7.3)		9.0(10.7)		130.0 (133.4)	
Institution								
Ambulatory (*n* = 259)	7.0 (7.4)	*p* = 0.005**	8.0 (8.0)	*p* = 0.440**	11.0 (10.7)	*p* = 0.230**	126.0 (129.0)	*p* < 0.001**
Hospital (*n* = 384)	3.0 (5.8)		7.0 (7.8)		9.0 (10.6)		130.5 (133.4)
Workload								
<0.5 full-time (*n* = 43)	12.0 (10.4)	*p* = 0.002***	12.0 (11.0)	*p* < 0.001***	15.0 (13.4)	*p* < 0.001***	128.0 (132.4)	*p* = 0.884***
0.6–1.0 full-time (*n* = 395)	3.0 (5.9)		6.0 (7.1)		9.0 (9.7)		129.0 (130.8)	
1.1–1.5 full-time (*n* = 205)	5.0 (6.7)		8.0 (8.7)		11.0 (11.8)		127.0 (133.3)	
One workplace								
Yes (*n* = 541)	3.0 (6.3)	*p* = 0.016**	7.0 (7.8)	*p* = 0.187**	10.0 (10.6)	*p* = 0.374**	128.0 (130.9)	*p* = 0.507**
No (*n* = 102)	6.0 (7.4)		8.0 (8.3)		12.0 (11.0)		127.5 (135.6)	
Work schedule								
Day shift (*n* = 336)	5.0 (6.7)	*p* = 0.005***	7.0 (7.6)	*p* = 0.025***	10.0 (10.0)	*p* = 0.002***	127.0 (130.7)	*p* = 0.022***
Night shift (*n* = 19)	12.0 (10.6)		8.0 (9.9)		11.0 (12.3)		125.0 (123.4)	
24-h shifts (*n* = 83)	2.0 (7.8)		8.0 (9.7)		15.0 (12.7)		132.0 (130.1)	
Mixed shifts (*n* = 205)	3.0 (5.0)		7.0 (7.4)		9.0 (10.6)		130.0 (134.7)	

* Spearman correlation coefficient; **Mann–Whitney U test; ***Kruskal-Wallis H test.

Higher job satisfaction was reported by those working for less than 1 year or more than 31 years in the same workplace, in hospitals, or on 24-h or mixed schedules (Table 2).

### 3.1 The associations between nurses’ depression, anxiety, stress, and job satisfaction

It was found that nurses’ depression was statistically significantly and moderately positively correlated with their stress (*ρ* = 0.633, *p* < 0.001) and strongly positively correlated with anxiety (*ρ* = 0.798, *p* < 0.001). Anxiety in nurses was also statistically significant and strongly positively correlated with their stress (*ρ* = 0.748, *p* < 0.001). Total job satisfaction was weakly negatively correlated with depression (−0.276, *p* < 0.001), moderately- with anxiety (−0.354, *p* < 0.001), and stress (−0.332, *p* < 0.001).

Additionally, using the Spearman correlation coefficient, it was determined that nurses’ depression, anxiety, and stress were statistically significantly negatively correlated with most of the job satisfaction facets. Depression was statistically significantly and very weakly negatively correlated with job satisfaction regarding contingent rewards (*ρ* = −0.149, *p* < 0.001), promotion opportunities (*ρ* = −0.138, *p* < 0.001), and work conditions (*ρ* = −0.127, *p* = 0.001); weakly negatively correlated with job satisfaction regarding supervision (*ρ* = −0.340, *p* < 0.001), the nature of the work (*ρ* = −0.335, *p* < 0.001), satisfaction with coworkers (*ρ* = −0.220, *p* < 0.001), and communication (*ρ* = −0.217, *p* < 0.001).

Anxiety was statistically significantly and very weakly negatively correlated with satisfaction with work conditions (*ρ* = −0.106, *p* = 0.007); weakly negatively correlated with satisfaction regarding supervision (*ρ* = −0.368, *p* < 0.001), the nature of the work (*ρ* = −0.332, *p* < 0.001), coworkers (*ρ* = −0.310, *p* < 0.001), contingent rewards (*ρ* = −0.224, *p* < 0.001), communication (*ρ* = −0.214, *p* < 0.001), and promotion (*ρ* = −0.208, *p* < 0.001).

Stress was statistically significantly and very weakly negatively correlated with satisfaction with work conditions (*ρ* = −0.131, *p* = 0.001), fringe benefits (*ρ* = −0.097, *p* = 0.014), and salary (*ρ* = −0.096, *p* = 0.015); weak negatively correlated with promotion (*ρ* = −0.314, *p* < 0.001), supervision (*ρ* = −0.272, *p* < 0.001), contingent rewards (*ρ* = −0.234, *p* < 0.001), coworkers (ρ = −0.228, *p* < 0.001), the nature of the work (*ρ* = −0.221, *p* < 0.001), and communication (*ρ* = −0.203, *p* < 0.001).

**Figure 2:**
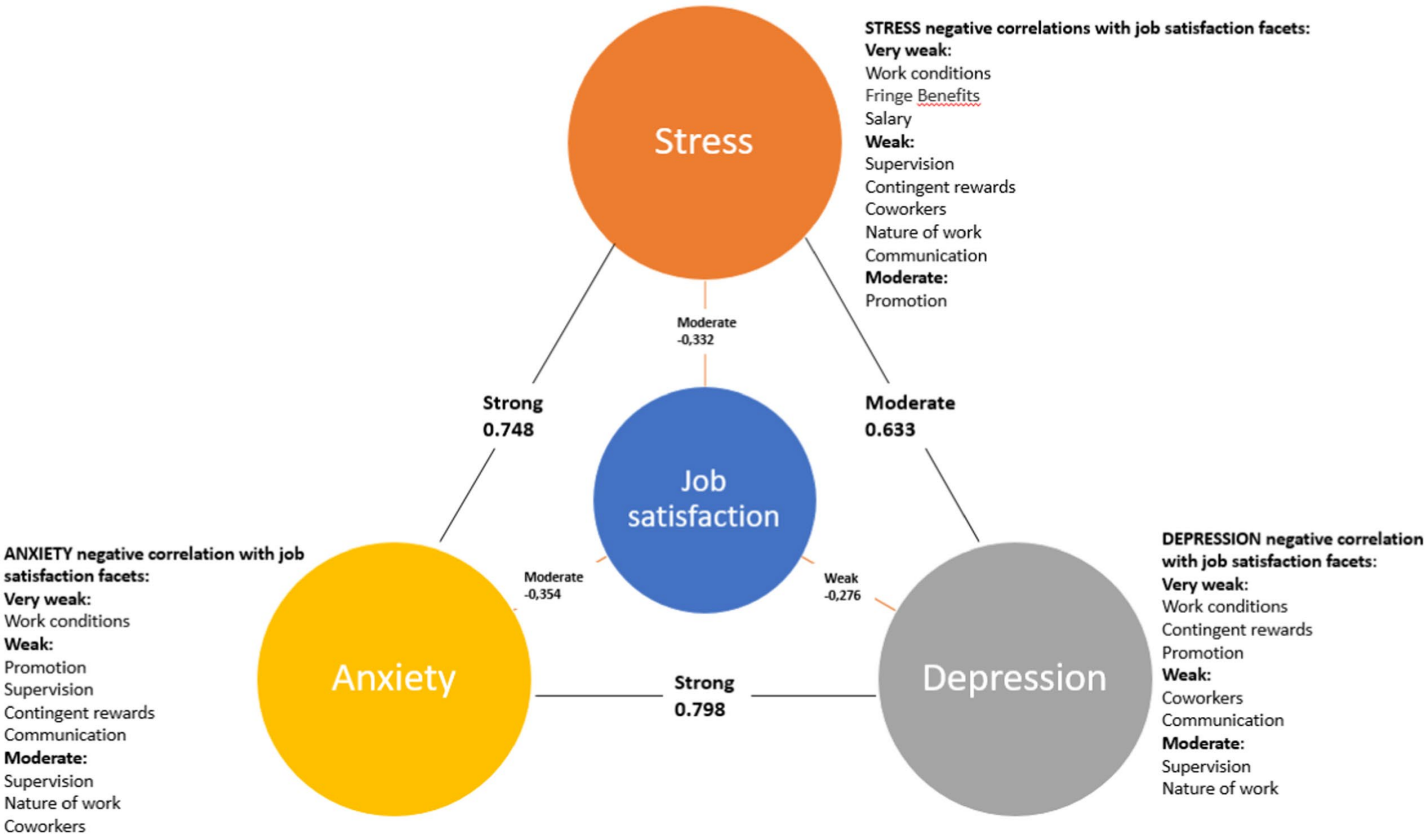
The associations between depression, anxiety, stress, and job satisfaction.

Figure 2 shows a summary diagram of the reliable correlations between psychoemotional state and job satisfaction as well as its facets:

## 4 Discussion

The results of this study provide valuable insights into the psychoemotional state and job satisfaction of nurses, shedding light on the complex relationships between these factors and various sociodemographic characteristics. None of the psychoemotional scales indicated a clinical threshold for a disorder, which suggests that while psychoemotional concerns exist, they are not at crisis levels for the majority of respondents. While the overall psychoemotional state of nurses was within normal limits, a significant portion faced deteriorating mental health. Nearly one-third of respondents reported experiencing stress (27%) or depression (30%), while nearly half (47%) indicated increased levels of anxiety. Among the nurses surveyed, 14.8 out of every 100 exhibited high or very high anxiety levels, 2.3 out of 100 showed severe depressive symptoms, and 1.4 out of 100 reported significant stress. Although we observed lower levels of depression and stress compared to the pandemic period, anxiety levels were found to be higher. A previous meta-analysis evaluating the psychoemotional state of healthcare professionals during COVID-19 reported the prevalence of depression, anxiety, and stress at 37.12, 41.42, and 44.86%, respectively (Mahmud et al., 2021). The prevalence of depression observed was similar to that reported in France as clinical depression in the post-COVID-19 era (Fond et al., 2022). Nursing is regarded as a profession with a high-stress risk, accounting for 50–80% of psychosomatic diseases or conditions (Luan et al., 2017), mainly due to the fast-paced nature of the work and the constant demand to manage emergencies (Schneider-Matyka et al., 2023).

Our study identified a weak to moderate relationship between psychoemotional state and job satisfaction. Similarly, a meta-analysis highlighted that emotional exhaustion, burnout, stress, depersonalization, depression, and anxiety all demonstrated modest but significant negative correlations with job satisfaction (Maqbali et al., 2024). Furthermore, Portero de la Cruz et al. (2020) described a significant negative correlation between stress and job satisfaction (*p* = 0.0004). In addition, it was found that a lack of social support was significantly associated with a higher risk of burnout, job dissatisfaction, depression, anxiety, and intention to quit (Norful et al., 2024). Also, Ambani et al. (2020) discovered that more than 80% of public hospital nurses reported high levels of burnout, 65% were dissatisfied with their job, and 33% intended to leave. However, another study showed that nurses working in university hospitals with suitable working environments had significantly lower rates of job dissatisfaction, burnout, and intention to leave (Nantsupawat et al., 2017).

Moreover, nurses experiencing stress, anxiety, or depression were more likely to worry about a lack of job satisfaction and conflicts with supervisors. Nurses identified different stressors in the workplace: nurses working in a private hospital identified job dissatisfaction conflicts with doctors and patients as stressors; in a public hospital, infectious diseases; in intensive care units, inadequate pay; in non-intensive care units, smells and sounds in the workplace, and conflicts with patients as stressors (Kaushik et al., 2021). Modaresnezhad et al. (2021) conducted a study that found that difficulties at work, work–family conflicts, and negative attitudes toward the team lead to anxiety, which reduces job satisfaction and ultimately leads to increased employee turnover. They identified supervisor support as one of the best factors that weakened the relationship between job dissatisfaction and turnover (Modaresnezhad et al., 2021).

Additionally, Wei et al. (2023) analyzed the mental health of surgical system nurses and job stress. The mental health of surgical nurses is influenced by workload, age, seniority, and job title. These factors should be considered in psychological interventions for operating theater nurses to improve the health of clinical nurses (Wei et al., 2023).

Interestingly, significant correlations were found between psychoemotional states and several sociodemographic factors. Higher levels of depression were linked to nurses working in outpatient settings, multiple part-time jobs, and night shifts. These findings are consistent with existing research indicating that nurses working in challenging environments or with irregular shifts may experience heightened stress and dissatisfaction (Jin et al., 2023; Quesada-Puga et al., 2024). It suggests that work environments with higher demands or night shifts may contribute to mental health struggles among healthcare workers.

The study also found that anxiety was more prevalent among younger nurses, males, those with less than 5 years of experience, or those working part-time or night shifts. These factors may reflect the challenges less experienced nurses face or those still adjusting to the profession’s demands. A lack of skills or confidence, combined with the uncertainty of fluctuating workloads, especially during night shifts can create significant tension and stress (Kisanuki et al., 2024).

Stress also was more pronounced in nurses with 1–5 years of experience or those working part-time and 24-h shifts. These results suggest that nurses in the early years of their careers, particularly those with a limited work-life balance, may be at a higher risk of experiencing psychoemotional strain.

Compared to job satisfaction norms in medical samples (Spector, 1985), our study revealed slightly higher overall satisfaction scores (131 vs. 129), as well as higher ratings for promotion (13.2 vs. 11.4), contingent rewards (13.7 vs. 12.9), and communication (15.5 vs. 14.4). Similar scores were observed for benefits (13.5 vs. 13.4), coworkers (16 vs. 16.8), operating procedures (13.8 vs. 13), and salary (12 vs. 11.8). However, slightly lower scores were noted for the nature of work (17.5 vs. 18.3) and supervision (16.6 vs. 17.4). The highest-rated facets were consistent with the norms: nature of work and supervision. In contrast, the lowest-rated facets were pay and promotion. A systematic review found that interpersonal relationships, working conditions, and recognition were the most frequently reported factors influencing nurse job satisfaction. Various extrinsic, intrinsic, personal, emotional, and psychosocial factors were also identified as significant contributors (Hudays et al., 2024).

When examining job satisfaction, the results revealed that higher satisfaction was associated with nurses who had worked at the same workplace for less than a year or more than 31 years. This paradox may reflect early-career nurses’ different needs and expectations versus those with extensive experience. New nurses may experience a sense of excitement or fulfillment from their initial professional experiences. At the same time, those with long tenures may feel more established or satisfied with their routine, possibly due to familiarity rather than new challenges. Scheduling patterns significantly influenced job satisfaction, with higher satisfaction observed among those working 24-h or mixed schedules.

A particularly noteworthy finding was the strong correlations between depression, anxiety, and stress. According to other authors, depression, anxiety, and stress often contribute to poor overall mental health, forming a complex network of interactions where they can mutually reinforce one another (Mofatteh, 2020). Depression was positively correlated with both anxiety and stress, reinforcing the interrelated nature of these psychoemotional states. Nurses experiencing high levels of stress were also more likely to experience anxiety, which, in turn, was negatively correlated with several components of job satisfaction, such as leadership, working conditions, and communication. Our findings align with others, showing that stress in nurses negatively affects care rationing and job satisfaction, with factors like gender, employment type, and workplace influencing stress levels (Schneider-Matyka et al., 2023). As stated by other authors, nurses often face excessive responsibilities, which hinder their ability to provide equal care. Work overload leads to rushed care, limited patient interaction, and rationed services, further aggravated by insufficient support, low wages, and limited opportunities for career advancement (Baka and Derbis, 2013). A mood-congruent theory highlights the profound impact of emotional states on cognitive processes, shaping how individuals perceive, recall, and assess their surroundings (Mayer et al., 1992). Positive emotions foster favorable evaluations and constructive behaviors by activating positive mental schemas, whereas negative emotions amplify attention to adverse information, leading to more critical judgments and behaviors. This dynamic underscores the importance of understanding emotional states in contexts where perception and decision-making play pivotal roles (White et al., 2018). These findings suggest that addressing mental health issues among nurses could significantly improve their overall job satisfaction (Søvold et al., 2021). Stress and anxiety may diminish nurses’ overall engagement with work-related factors, further emphasizing the need for systemic changes to improve work environments, such as better leadership, clear communication, and opportunities for professional development.

The negative correlations between psychoemotional states and job satisfaction highlight the critical role that mental health plays in job satisfaction (Zhang et al., 2020; Ma et al., 2020). Nurses reporting higher levels of depression, anxiety, or stress were less satisfied with various aspects of their work, including leadership, promotion opportunities, and incentives. These results are consistent with research showing that poor mental health often leads to lower job satisfaction (Steele et al., 2020), which, in turn, can affect job performance and retention. Errors in clinical practice were likely to happen during clinical practice due to nurses’ negative experiences (Pappa et al., 2023). The psychological burden of a nursing career is often intensified by persistent stigma and punitive regulatory practices, which discourage nurses from admitting mistakes and seeking help for mental health issues or substance use disorders (Choflet et al., 2021). It underscores the need for healthcare institutions to address nurses’ mental health needs and ensure that work environments are supportive, provide adequate rewards, and offer growth opportunities. By improving the work environment, nurses can experience improved psychological wellness (Lenzo et al., 2021). An organizational culture that values healthcare workers’ health and wellbeing has been found to improve their wellbeing and safety (Cunningham et al., 2024).

It is important to recognize that prolonged, uncontrolled stress can exhaust the psyche and weaken the immune system, often leading to the development of various illnesses (Tsigos et al., 2020; Yaribeygi et al., 2017). Anxiety and depression are among the most common mental states (World Health Organization, 2024; Institute of Health Metrics and Evaluation, 2023) associated with stress (Chu et al., 2022), significantly impacting physiological balance and affecting both physical and mental health (Palinkas and Wong, 2020).

There are several ways to decrease depression and anxiety among nurses. According to Liu et al. (2023), mindfulness-based intervention can be used as an effective way to reduce nurses’ anxiety and depression. They identified that the effect of an 8-week intervention was better compared to a 4-week intervention (Liu et al., 2023). Additionally, Melnyk et al. (2020) also discovered that mindfulness and cognitive-behavioral therapy-based interventions are effective in reducing stress, anxiety, and depression. Moreover, brief interventions with deep breathing, visual triggers, pedometers, and health coaching may be beneficial (Melnyk et al., 2020).

Several limitations to this study should be noted. Firstly, the research was conducted in a specific geographical location (Klaipėda County, Lithuania), so the generalizability of the results to other regions is limited. Different healthcare structures and working environments may have influenced the results. Further research with more diverse samples could help strengthen the external validity of the findings. Additionally, a longitudinal study would provide additional information about the topic. A relevant sample of nurses was included in the study. However, due to the non-probability purposive sampling method, the sample’s representativeness is limited.

## 5 Conclusion

The findings highlight that while average levels of depression, anxiety, and stress among nurses remain within normal limits, a substantial proportion faces notable mental health challenges, particularly anxiety. Factors such as workload, work schedule, and job experience significantly impact their psychoemotional state. The strong interrelation between depression, anxiety, and stress underscores the need for comprehensive mental health support in the workplace. Moreover, the negative association between mental health and job satisfaction indicates that improving nurses’ working conditions and wellbeing could enhance their mental health and professional satisfaction. The findings suggest that healthcare organizations need to pay greater attention to the mental health of their nursing staff and work toward creating environments that support both psychological wellbeing and job satisfaction. Reducing stress, addressing anxiety, and providing better leadership and career development opportunities may help improve nurses’ mental health and, consequently, their overall job satisfaction. Addressing these challenges is essential to sustaining a healthy and productive nursing workforce and improving job performance and competitiveness.

## Data Availability

The raw data supporting the conclusions of this article will be made available by the authors, without undue reservation.
